# Tracking COVID-19 by Tracking Infectious Trajectories

**DOI:** 10.1109/ACCESS.2020.3015002

**Published:** 2020-08-07

**Authors:** Badreddine Benreguia, Hamouma Moumen, Mohammed Amine Merzoug

**Affiliations:** Computer Science DepartmentUniversity of Batna 2 Batna 05078 Algeria

**Keywords:** Big data, coronavirus, COVID-19, infection tracking, information and communications technologies, Internet of Things

## Abstract

Nowadays, the coronavirus pandemic has and is still causing large numbers of deaths and infected people. Although governments all over the world have taken severe measurements to slow down the virus spreading (e.g., travel restrictions, suspending all sportive, social, and economic activities, quarantines, social distancing, etc.), a lot of persons have died and a lot more are still in danger. Indeed, a recently conducted study [1] has reported that 79% of the confirmed infections in China were caused by undocumented patients who had no symptoms. In the same context, in numerous other countries, since coronavirus takes several days before the emergence of symptoms, it has also been reported that the known number of infections is not representative of the real number of infected people (the actual number is expected to be much higher). That is to say, asymptomatic patients are the main factor behind the large quick spreading of coronavirus and are also the major reason that caused governments to lose control over this critical situation. To contribute to remedying this global pandemic, in this article, we propose an IoT^*a*^ investigation system that was specifically designed to spot both undocumented patients and infectious places. The goal is to help the authorities to disinfect high-contamination sites and confine persons even if they have no apparent symptoms. The proposed system also allows determining all persons who had close contact with infected or suspected patients. Consequently, rapid isolation of suspicious cases and more efficient control over any pandemic propagation can be achieved.

## I. Introduction

In December 2019, a novel virus has emerged in Wuhan city. This disease, named coronavirus or COVID-19, has quickly spread throughout China and then to the entire world. As of July 2020, more than 200 countries and territories have been affected and over 15 million people have been diagnosed with the virus. Since no cure or vaccine has been found and since tests cannot be applied on a large scale to millions of persons, governments had and still have no choice but to take severe actions such as border closing, travel canceling, curfews, quarantines, and contact precautions (facemasks, social distancing, and self-isolation). The authorities have also implemented strategies that aim to rapidly detect infections using different cutting-edge medical tools (such as thermal cameras, blood tests, nasal/throat swabs, etc.). On the one hand, these measurements, which for the time being constitute the only possible solution, have succeeded to slow down the contagion spreading, but on the other hand, they have also caused considerable economic damages, especially to countries with brittle economies (suspension of all sorts of activities: social, economic, educational, etc.).^a^IoT: Internet of Things.

**FIGURE 1.**
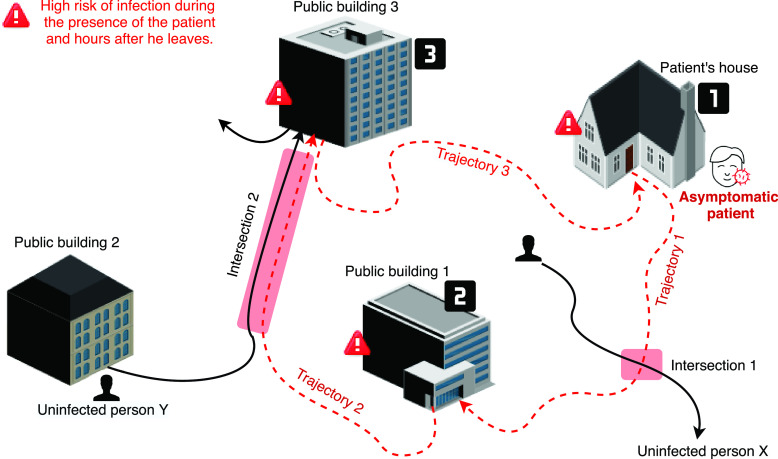
Infection tracking - example of historical trajectories taken by a COVID-19 patient and all persons (resp. places) who he might have encountered/infected (resp. visited).

The rapid spreading of coronavirus is due to the continuous person-to-person transmission [2]–[4]. In addition to this, a recent study has also suggested that a second factor is playing a major causal role in this high virus spreading; namely the *stealth transmission* [1]. The coronavirus can take 14 days before the appearance of symptoms. During this incubation period, asymptomatic patients, called *undocumented patients*, can infect large communities of people. In turn, these newly infected persons, who will remain unaware of their illness (until they eventually develop the symptoms), can also infect larger communities, thus, leading to an uncontrollable domino effect [1], [5], [6]. Accordingly, to confine and effectively eliminate the coronavirus, it is crucial and mandatory to possess an efficient investigation system that allows determining (1) highly infectious places and (2) all the persons who were in contact with patients who have recently tested positive. Indeed, persons who are known to be in direct relationship with a patient (such as family members, friends, and coworkers) can be easily determined and tested. However, numerous other persons could have also been in contact with this infected individual. All these persons, who cannot be easily determined, can contribute to the widespread of the virus. To remedy this issue, in this article, we propose an IoT investigation system that can determine the exact trajectories of infected persons (exact coordinates labeled with time). Hence, as shown in Figure 1, the proposed solution allows determining areas of high contamination and also provides a quick detection mechanism of undocumented patients who are known to be the main and most important factor in the rapid widespread of coronavirus. These persons (resp. places) can be tested/confined even if they had no blatant symptoms (resp. closed and properly disinfected).

More in detail, Figure 1 illustrates an example of a recorded patient trajectory (presented by red dashed lines/arrows: *trajectory_1*, *trajectory_2*, and *trajectory_3*). Note that due to the viral transmission property, both individual $X$ and $Y$ (who have intersected paths with the asymptomatic patient in, respectively, *intersection_1* and *intersection_2*) are suspected to be infected. Also, public buildings that have been visited by this patient (i.e., *public building_1* and *public building_3*) are considered as potential areas of transmission.

The investigation system presented in this article is a proposition to governments and lawmakers. So, it needs to be approved and adopted only in the case of highly dangerous pandemics and public health emergencies. In summary, to properly operate, the proposed system must be continuously fed with the coordinates of persons who are in public crowded places (mainly using IoT devices that can identify persons and report their locations). Although this proposed solution can open large debates from the standpoint of privacy and human rights, during extreme critical situations in which the entire human race might be at stake, the urgency of saving lives becomes of higher priority. In such circumstances, the proposed technique can be quickly applied as a last resort by higher authorities (governments, WHO,^1^ etc.) while giving guarantees about (1) protecting the privacy of people, and (2) disabling this system once the outbreak is contained.^1^WHO: World Health Organization.

The remainder of this article is organized into six sections as follows. Section II describes and details the proposed system. Section III presents a small-scale implementation example of this proposal. Section IV addresses the benefits that the proposed investigation system can bring to state-of-the-art mathematical disease spreading/prediction models. Section V discusses the main advantages and shortcomings of the proposed solution. Section VI provides a brief review of existing tracking tools (related work). Finally, Section VII concludes the paper and provides some recommendations.

## II. Proposed Solution

In the proposed system, a big-data architecture is considered to archive the continuously collected trajectories of persons. This archiving structure, which is inspired by the big data model proposed in [7], must (as previously mentioned) be fed by IoT devices that can (1) determine coordinates of persons during their outdoor activities and (2) send the collected data to the system. We point out that persons staying at home or in their vehicles are assumed to be isolated (i.e., they cannot infect other persons nor they can be infected). Consequently, they do not need to activate the coordinates collection process. On the contrary, to ensure their safety by ensuring the proper operation of the system, it is necessary for any person leaving his/her house or car to activate coordinates collection and save his/her tracks.

**FIGURE 2.**
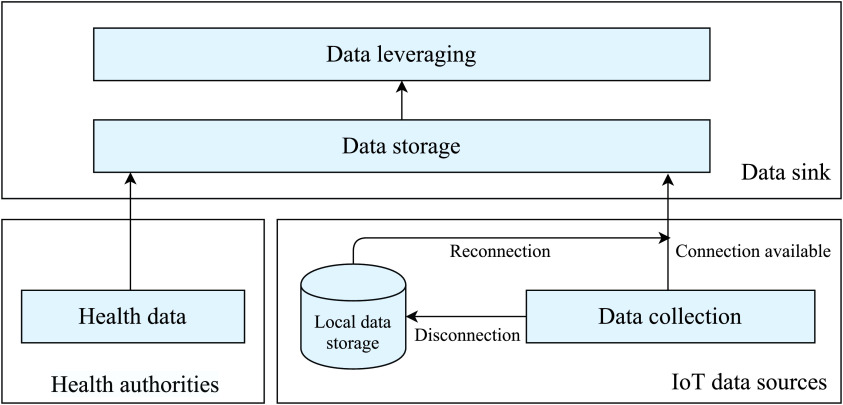
Overall system architecture.

As Figure 2 depicts, the proposed architecture has three main basic layers; namely, data collection, data storage, and data leveraging. In the remainder of this section, we describe each of these layers and provide their specific complementary tasks.

### A. Data Collection

As shown in Figure 2, the data gathering process is divided into two parallel independent tasks. Whereas the first one is related to collecting the necessary health data, the second is responsible for collecting the geolocalization coordinates of persons.

#### 1) Health Data

Reporting the coronavirus deaths and all the newly confirmed and recovered cases is a basic stepping stone in the overall investigation process. For instance, once a new case has been discovered, the latter must be immediately reported to the automatic investigation system to allow it to find all the individuals who could have been infected by this person. In the same context, given the fact that patients are the main factor in the system, once some of them have recovered from the virus, their statuses must be immediately changed from active to recovered, and so forth.

On the contrary to the second task (i.e., geolocalization data gathering) in which data is automatically captured and reported by the employed IoT devices, in this first task, data must be manually entered by health authorities. For example, hospitals can be responsible for providing the newly infected cases, and all the other indispensable updates.

#### 2) Geolocalization Data

In literature, there exist a plethora of techniques that allow the determination of device geographic locations. The Global Positioning System (GPS) is the most widely known and used technique on mobile devices (such as smartphones, smartwatches, vehicles, etc.). However, given the fact that GPS can be sometimes inaccurate (in the order of dozens of meters, especially in cities), other techniques, proposed in the literature, can be considered. Among these solutions, we mention, radio-frequency, cellular networks, sensor networks, or even Internet-based localization techniques [8]–[12]. These approaches can accurately estimate device locations (for both indoor and outdoor scales like buildings and cities, etc.), but they must be adapted for public wide usage.

Indeed, since GPS technology is freely available on smartphones, smartwatches, etc., it is more convenient to utilize it for tracking-systems destined to controlling outbreaks and stopping infection propagations. In other terms, each mobile device with a GPS receiver can be programmed to capture its coordinates and contribute to the global tracking process. For instance, nowadays, it is possible to design a bracelet/device using simply: (1) an Arduino module, (2) GPS receiver, and (3) Wi-fi component. The operation principle of this device can consist of periodically detecting its geolocalization coordinates and locally saving them. Once the device is connected through Wi-fi, the collected coordinates can then be sent to the designated global destination.

More generally, different approaches can be considered to collect the coordinates of persons who left their houses. For example, (1) relying on telecom and IT infrastructures, (2) equipping public buildings with appropriate devices, or (3) utilizing dedicated tracking apparatuses.

• *Telecom/high-tech companies:* using telecommunication technologies, smartphones can be easily instructed (programmed) to periodically report their actual location to a dedicated storage system (phone-number, GPS-coordinates, time).
• *Public buildings:* by installing face recognition cameras on the entries/exits of public buildings, the identity of visitors can be easily determined and then sent to the designated infrastructure (person-id, building-id, entry-time, exit-time). This way, based on the information provided by the different public buildings and facilities, the tracks of infected people can be easily determined. The following lines provide an example of places that were visited by an infected person P1: (P1, building, (entry, T1), (exit, T2)), (P1, shopping mall, (entry, T3), (exit, T4)),(..,..,(..,..),(..,..)), (P1, airport A, (entry, Tm), (exit to plane, Tn)), (P1, airport B, (entry from plane, Tx), (exit, Ty)), (..,.., (..,..), (..,..).
  The main drawback of this second data collection approach is that it cannot be easily implemented in most countries due to the lack of appropriate underlying identification/recognition systems. However, this solution can be utilized in the case where authorities fail to convince people of using their cellphones as tracking devices.
• *Specific solutions and electronic devices*: the two aforementioned approaches can be adopted independently or in combination. Actually, during dangerous outbreaks where rapid tracking of infected people becomes an urgency, in addition to personal smartphones and public indoor (entry/exit) cameras, governments can consider using other tracking techniques like for instance electronic bracelets, public outdoor security cameras, drones, or even satellites. Furthermore, to consolidate the previous two approaches, solutions such as Bluetooth or NFC^2^ can also be considered [8]–[12]. In the same context, as previously mentioned, in the event of technical issues related to GPS, governments can opt for alternative efficient geo-localization techniques [9], [11].

^2^NFC: Near-Field Communication.

As their name implies, the IoT devices (responsible for collecting person tracks) report the required data using the Internet (via wired or mobile wireless networks: 4/5G,...). So, as shown in Figure 2, to avoid losing important information in the case of Internet disconnection, each device must store locally the collected data. Once reconnected, these IoT devices can then send the gathered data to the system. Finally, it is worth mentioning that to ensure the success of the whole virus tracking process, IT companies, public institutions, and third-parties must contribute to collecting and reporting the required data.

### B. Data Storage

Big data, which refers to enormous datasets, has five essential characteristics known as the 5 Vs: volume (data size), velocity (data generation frequency), variety (data diversity), veracity (data trustworthiness and quality), and finally value (information or knowledge extracted from data). To address the issues related to storing and processing huge data volumes, extensive research has been done and numerous efficient solutions have been proposed (data ingestion, online stream processing, batch offline processing, distributed file systems, clustering, etc.). These techniques provide both efficient distributed storage and fast processing. Currently, research is more focused on big data leveraging (i.e., the last V which consists of turning data into something valuable using data analytics, machine learning, and other techniques) [7], [13]. This point, which represents the main contribution of our work, will be detailed in the next subsection.

As stated in [7], an ideal big data storage/processing infrastructure must adhere to the four following major requirements: (1) time and space efficiency, (2) scalability, (3) robustness against failures and damages, and (4) data security/privacy. For instance, regarding this last point, since the collected data in our context is personal and highly sensitive, strict laws and regulations must be imposed to protect it. The established privacy model must determine who can access data (governments, higher health authorities, WHO organization, etc.), under what constraints, for how long data can be kept, and to whom this data can be distributed, etc. Moreover, in addition to data privacy, all conventional data security mechanisms must also be considered (confidentiality, integrity, and availability).

To ensure the proper operation of the system, data that is continuously coming from the various considered IoT sources (such as sensors, smartphones, cameras, bracelets, etc.) must provide three main information: *person-id*, *coordinates*, and *time* (Table 1). The received trajectories of persons are recorded as an immutable discrete set of points (coordinates) labeled with time. For example, according to Table 1, the recorded traces of person P1 along with their corresponding time instants are: (c1, t1), (c4, t3), (c5, t4), (c2, t5), and (c3, t6). Note that in Table 1, for any given person, time is a monotone function that must keep growing (it cannot go back). Note also that more than one person can exist in the same coordinates at the same time. For example, P1 with P3 at (c2, t5) and P1 with P5 at (c3, t6).

**TABLE 1:** Example of Collected Trajectories

Person-ID	Coordinates	Time
P1	c1	t1
P2	c2	t1
P3	c3	t2
P1	c4	t3
P1	c5	t4
P1	c2	t5
P3	c2	t5
P4	c6	t5
P5	c3	t6
P1	c3	t6
…	…	…

In the considered context, the *frequency* according to which data must be collected (i.e., the second V) has a direct significant impact on both the first and last Vs (i.e., *data size* and *data value*). High-quality trajectories can be achieved by increasing the frequency of data collection. This, however, can considerably increase the volume of data. In contrast, low collection frequencies reduce the data size but yield low trajectory quality. So, a tradeoff between the quality of trajectories and the size of data must be defined.

**FIGURE 3.**
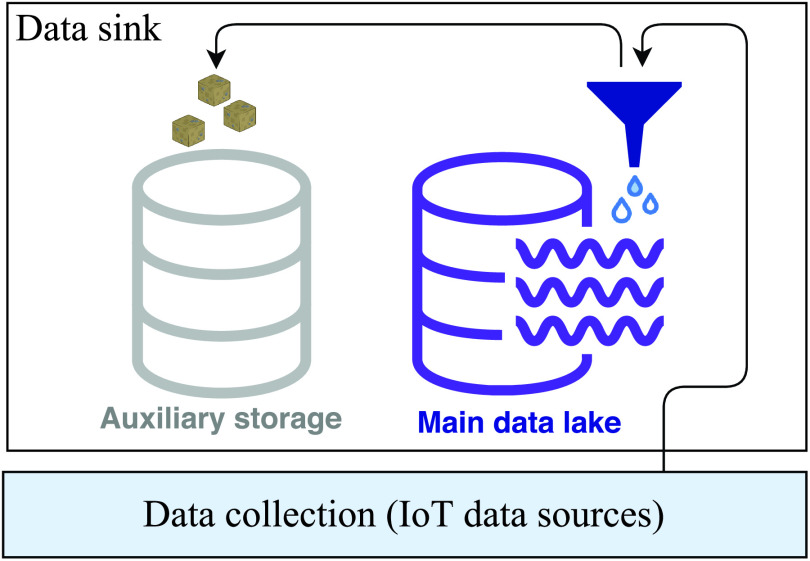
Data filtering (preprocessing).

The main goal of the proposed system is to track the main sources of infection, whether they would be humans or places. But, in reality, the quality of the collected data can have a deep impact on the system’s performance; it can contribute to its efficiency or deficiency. To prevent useless noisy data from harming or affecting the decisions of the investigation process, a filtering mechanism must be added (Figure 3). If it appears that the filtered data is useless, it can then be safely deleted. For example, data collected from highways and main roads is not of value. In this scenario, users are inside their respective cars and the risk of infection is nil (similarly to houses, people inside the same vehicle are considered as isolated). Concretely speaking, the responsibility of data filtering can be left to authorities, which can decide which geographic zones or areas are of interest.

Before moving to the next subsection II-C (Data leveraging), in the following, we will address some issues and points related to this last important operation (efficient data storage and quick query responses to both the authorities and users).

#### 1) Data Management

As we have previously mentioned, the large collected data needs important storing space (especially when considering large countries with tens or hundreds of millions of citizens). To do so, the numerous proposed big data products and technologies can be utilized to facilitate the process of storing, managing, searching, and extracting useful information.

Table 1 illustrates how the captured data can be stored. It can be seen as a data mine/lake with continuous growth. Note that each new collected person coordinates are recorded as a new row (*Person-ID*, *Coordinates*, *Time*). Thus, if we assume that $r$ is the size of one row in Bytes (a few dozens of Bytes), then each person needs at most $r \times n$ Bytes per day (to record its coordinates), where $n$ is the frequency of geolocation capturing.^3^ Accordingly, a country of 40 million citizens like Algeria needs 4 TB per day for rows of 70 Bytes and a data collection frequency of one minute.^3^Recall that some scenarios like staying at home or being inside a car do not require geolocation data collection.

With that being said, a suitable efficient data organization/structure must be considered to avoid straining the database. Moreover, queries, such as finding out with whom a certain person $X$ has met, are very costly in terms of research operations and time. Therefore, to accelerate the exploitation process, it is important to index data (according to time and/or locations). For instance, recall that indexing tables in conventional relational databases allows accelerating data access in logarithmic time with regards to the number of rows.

### C. Data Leveraging

In the proposed system, only two operations can be performed on the collected geolocalization data: *storage* and *leveraging* (Figure 2). This section presents our three proposed algorithms, which exploit the gathered data to (1) find and classify suspected cases (i.e., persons who met with confirmed patients or other suspected cases), (2) determine black areas (zones with high contamination probability), and (3) find all persons who visited black areas (also considered as probably infected persons). For the convenience of the presentation, Table 2 compiles all the variables utilized by the different proposed algorithms presented respectively in the following three subsections.

**TABLE 2:** Variables Used in the Proposed Algorithms

	Variable	Definition
Common	$(P_{i},c_{i},t_{i})$	A recorded tuple: person identity, coordinates, and the corresponding collection time.
	$Trajectory$	Set of all trajectories.
	$Patient$	Set of all confirmed patients. A patient is defined as $(P_{i},t_{i})$.
	$aux$	A boolean that indicates whether an end-user is a suspected case.
	$S$	Server side.
	$C$	Client side.
Algorithm of Figure 5	$SUSPECTED_{t}$	Set of all suspected persons at time $t$.
	$suspected_{t,d}$	Set of suspected persons at distance $d$ and time $t$.
	$d$	An integer that indicates how close/far is a person to a confirmed patient.
	$k$	An index to iterate through all distances.
	$CD$	Current date.
	$IP$	Incubation period.
Algorithm of Figure 7	$A$	Set of all areas $(a_{i}, ~c_{i})$.
	$BA$	Set of all black areas $(ba_{i}, ~c_{i})$.
	$count_{k}$	Number of patients who have been to area $k$.
	$\alpha $	A threshold to decide whether a location is a black area.
Algorithm of Figure 9	$SUSPECT_{t}$	Set of all suspected persons who have frequented black areas at time $t$.
	$BA$	Set of all black areas $(ba_{i}, ~c_{i})$.
	$suspect_{t,k}$	Set of suspected persons who have frequented black area $k$ at time $t$.
	$s$	An index to explore all black areas.

#### 1) Suspected Cases Determination

**FIGURE 4.**
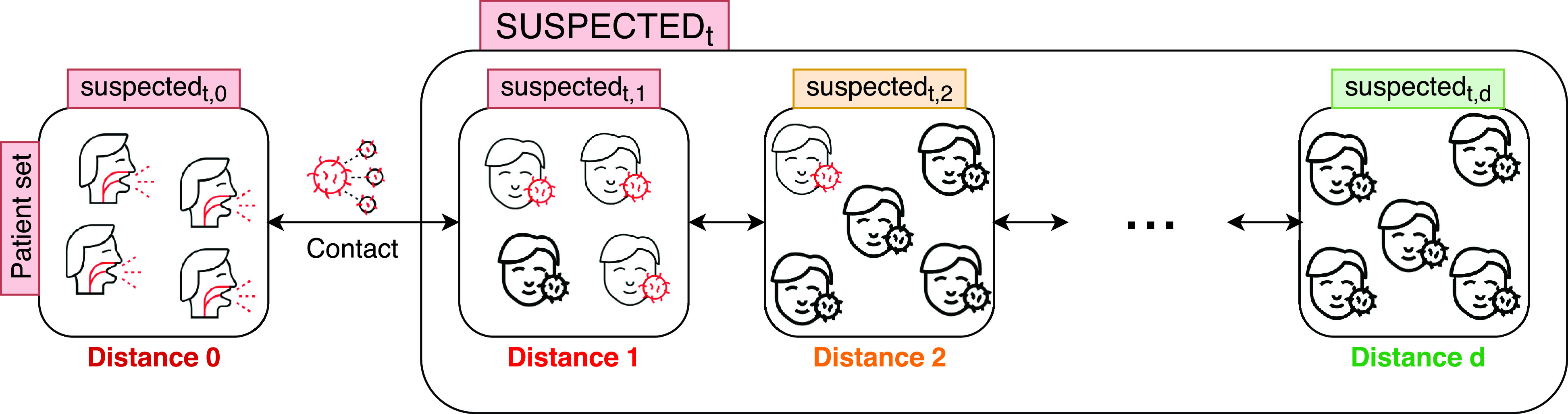
Set of all suspected persons at time t.

Based on the stored data (both recorded trajectories of people and health information), the main goal is to deduce all possible infections. As previously mentioned, the proposed investigation system does not only find the suspected cases but also categorizes them into several disjoint subsets denoted $suspected_{t, d}$ (Figure 4 and Table 2). The variable $d$, which stands for distance or degree, indicates how close/far is a person to a confirmed patient. The initial class of persons $suspected_{t, 0}$ represents the set of all patients (confirmed cases). If set $suspected_{t, 1}$ is defined ($suspected_{t, 1} \neq \phi $) then this means that it contains all individuals who had direct contact with at least a confirmed patient $P_{c}$ from $suspected_{t, 0}$ ($\forall ~P_{c} \in suspected_{t, 0}$). As regards the elements (persons) of $suspected_{t, 2}$, they had no direct intersection with a positive case from $suspected_{t, 0}$ (set of confirmed cases), but they have certainly met at least an element from $suspected_{t, 1}$. In general, for $i >= 2$, each element of $suspected_{t, i}$ has certainly met at least an element from $suspected_{t, (i-1)}$, but none from $suspected_{t, (i-2)}$.

**FIGURE 5.**
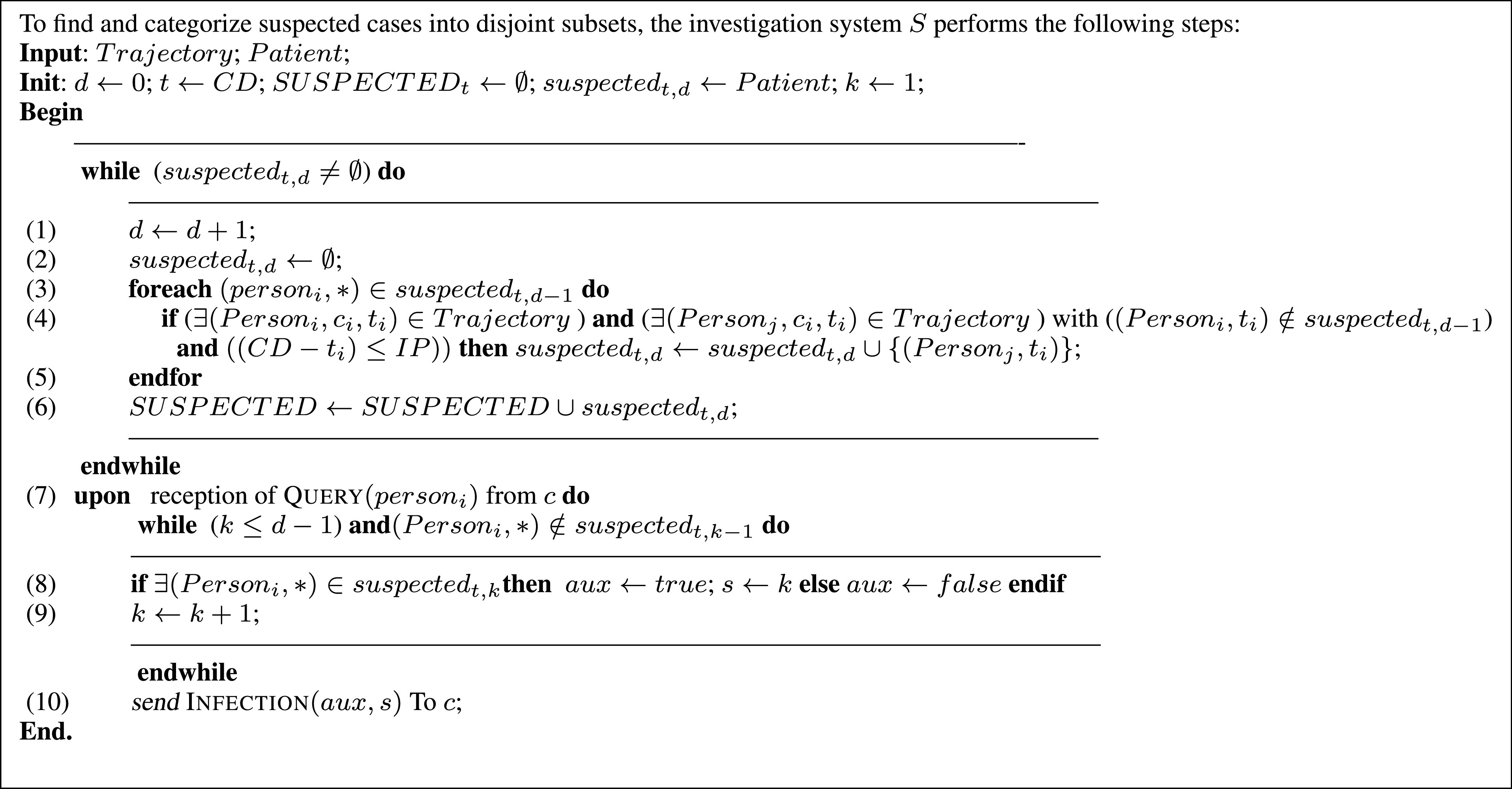
Main investigation algorithm - finding and categorizing suspected cases into disjoint subsets.

To properly operate, the proposed approach must be given the initial set of patients $suspected_{t, 0}$ (suspects at distance 0). As shown by the algorithm of Figure 5, at each iteration, based on the precedently entered/calculated set $suspected_{t, (i-1)}$, the new set of suspected persons $suspected_{t, i}$ is determined. Note that a person is considered to be suspected if he has met a patient or another suspected person during the incubation period (line 4: $(CD-t_{i})\leq IP$). The algorithm stops when the previously entered/calculated set $suspected_{t, (i-1)}$ is empty (which means that the next set of suspected persons $suspected_{t, i}$ cannot be calculated). Thus, after its execution, the proposed algorithm gives a classification (partition) of all individuals stored in the system: $suspected_{t, 0}$, $suspected_{t, 1},\ldots, suspected_{t, d}$ (SUSPECTED_*t*_ set). The remaining unclassified persons are considered uninfected. They have not met with confirmed patients from $suspected_{t, 0}$ nor with suspected persons from $suspected_{t, i}$ (with $i > 0$).

**FIGURE 6.**
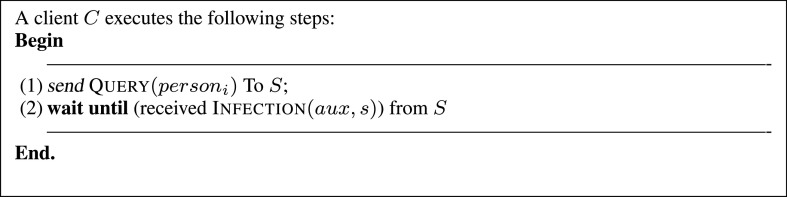
Algorithm executed by clients (e.g., mobile applications).

When a user (client) sends a query to the investigation system (Algorithm of Figure 6), he will receive to which class he belongs (i.e., $suspected_{t, 0}$, $suspected_{t, 1}$, $suspected_{t, 2}$,…or $negative$ category). Using the distance $d$ (rather than the identity of persons) allows informing (reassuring or alerting) users about their current statuses without exposing the privacy of other users of the system. The distance $d$ can be seen as a warning, the more a user’s distance is close to zero, the higher the risk of infection will be, and vice versa (Figure 4). Finally, we point out that in the proposed system, geolocalization data is periodically collected every $\Delta t$ period. To avoid any synchronization issue that might occur during this process, time differences that are less or equal to $\Delta t$ are ignored. For example, if a person $(P_{i}, ~c_{i}, ~t_{i})$ has the same coordinates as another person $(P_{j}, ~c_{j}, ~t_{j})$ and the difference between the collection instants ($t_{i}$ and $t_{j}$) of these coordinates ($c_{i}$ and $c_{j}$) is less or equal to $\Delta t$ then $t_{i}$ and $t_{j}$ will be seen as equal ($t_{i} = t_{j}$).

Moreover, as we have previously pointed out, GPS coordinates might be inaccurate. To deal with this kind of error, a threshold $\epsilon $ is used to tolerate coordinates differentiation ($\Delta c_{ij}$). That is, any two persons having coordinates $c_{i}$ and $c_{j}$ at time $t$ are considered in the same location if $\Delta c_{ij} < \epsilon $. Also, during a sufficient period where the virus transmission could take place between two persons (for example 15 minutes), the system captures coordinates many times, which allows masking GPS errors.

#### 2) Black Areas Determination

**FIGURE 7.**
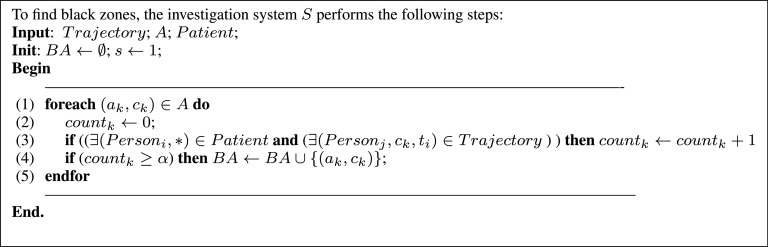
Black areas determination algorithm.

Another major problem that must be tackled is the possibility of virus transmission without direct human contact. This can happen, for instance, when an undocumented patient visits a public place and leaves the virus there (he touches objects found in that area, he sneezes, coughs, etc.). In such a scenario, as long as the virus stays alive, the probability of contamination remains very high. The algorithm of Figure 7 demonstrates the process followed to find all black areas. The key idea consists of determining if numerous patients have visited the same location. If so, then this zone is likely an infectious area of high viral transmission. More concretely, for each public location $a_{k} \in A$ (where $A$ is the predefined set of all public areas), the number of confirmed patients who have visited this area is determined using the $count_{k}$ variable. If this number overtakes the predefined threshold $\alpha $, the corresponding location $a_{k}$ is then considered to be a black area.

#### 3) Suspects From Black Areas

**FIGURE 8.**
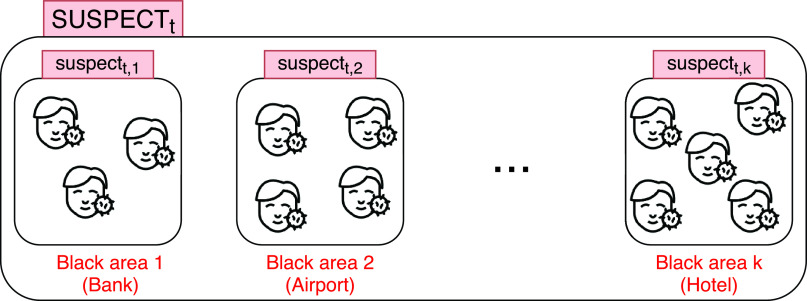
Set of all suspected persons who have frequented black areas at time t.

**FIGURE 9.**
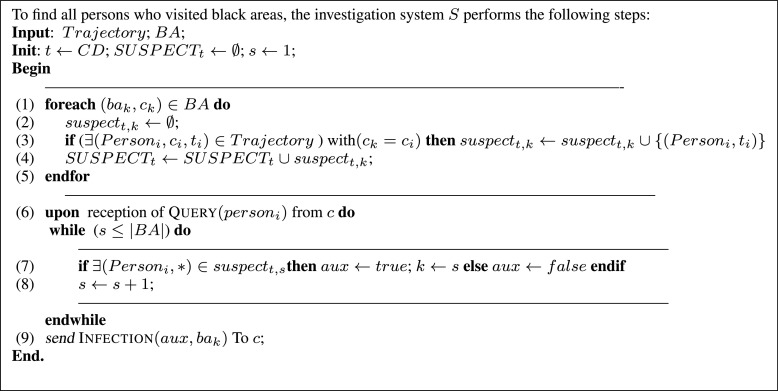
Algorithm for finding persons who have visited black areas.

As depicted in Figure 8 and Algorithm of Figure 9, once black areas have been determined, all persons who have been in these infectious zones can be straightforwardly found. More specifically, based on the set of captured trajectories, the proposed algorithm finds each person $P_{i}$ who has visited at least one black area $ba_{k}$. Each of these found suspected people will be added to the corresponding $suspect_{t, k}$ set (list of all suspected persons who have frequented black area $k$ at time $t$). In other terms, after its execution, the algorithm depicted in Figure 9 gives a different classification (partition) of all individuals stored in the system: $suspect_{t,0}$, $suspect_{t,1},\ldots, suspect_{t,k}$ (SUSPECT_*t*_ set). The unclassified persons are considered uninfected (they have not visited any infectious areas $ba_{k} \in BA$). Accordingly, the investigation system can be queried about black areas and the people who frequented them.

## III. System Implementation

**FIGURE 10.**
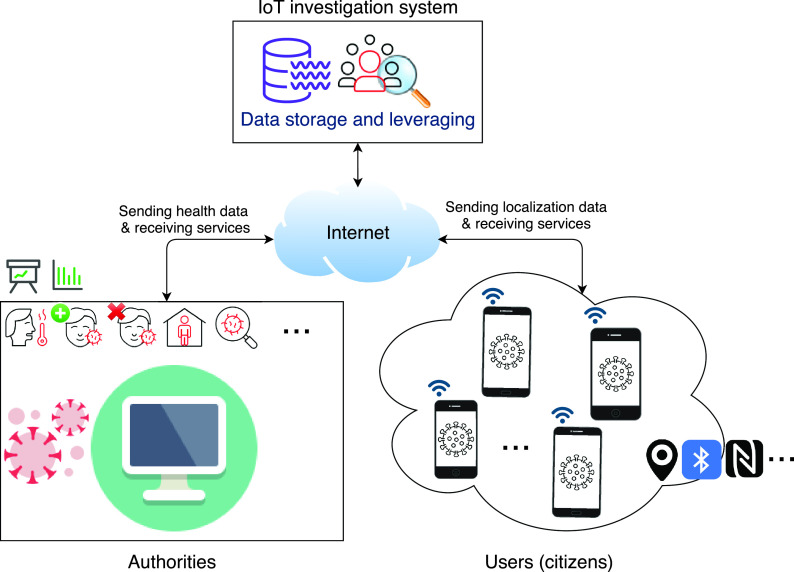
Developed application architecture.

This section describes the small-scale version of our proposed system that we have implemented. The main purpose of this demonstration is to show the applicability of the ideas discussed earlier in the paper. In brief, the implemented system has three essential parts (Figure 10): (1) mobile phone application for end-users, (2) automatic investigation mechanism (described in sections II-B and II-C), and (3) an interface dedicated to the government and health authorities.

### A. User Application

The developed mobile application collects the required geo-localization information by sending the GPS longitude and latitude coordinates along with the corresponding time to the investigation system. This operation is repeated periodically every $x$ unit of time. To avoid redundancies (useless data), during this defined period (i.e., $x$ time unit), when a user remains in the same location, his coordinates will not be reported to the system nor stored in the phone local database. Similarly, if a user moves for a certain distance of $y$ meters or less, he will be considered as stationary and his coordinates will not be collected. Also, as previously mentioned, in the event of Internet disconnection, each smartphone will store locally the gathered data and wait for reconnection.

**FIGURE 11.**
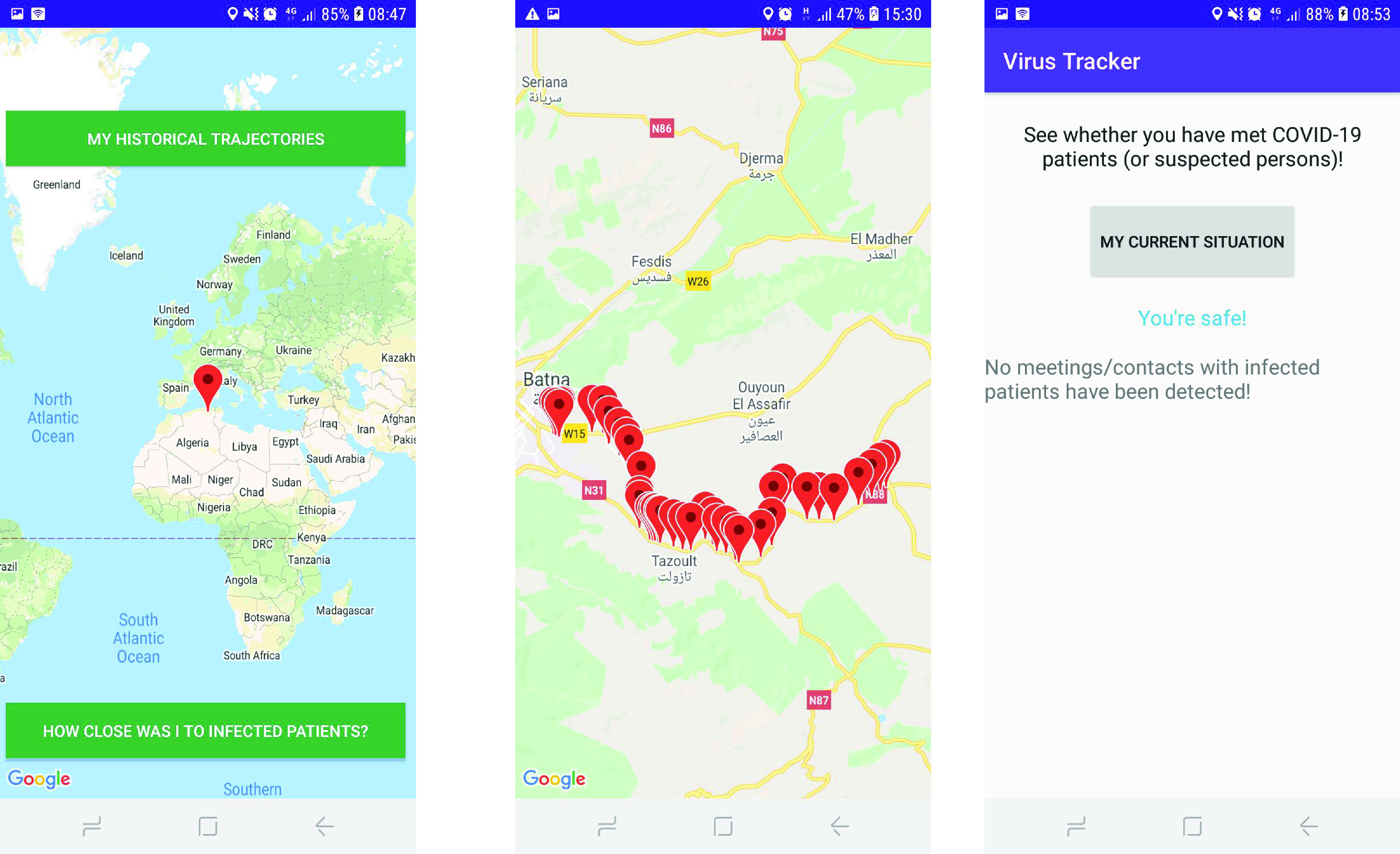
Screenshots from the developed application.

The application can offer numerous services to the end-users. For instance, users can review their historical trajectories during any possible correct period that they can define (Figure 11). A second most important service that can also be offered is allowing end-users to know if they have met infected people.

### B. Investigation System

**FIGURE 12.**
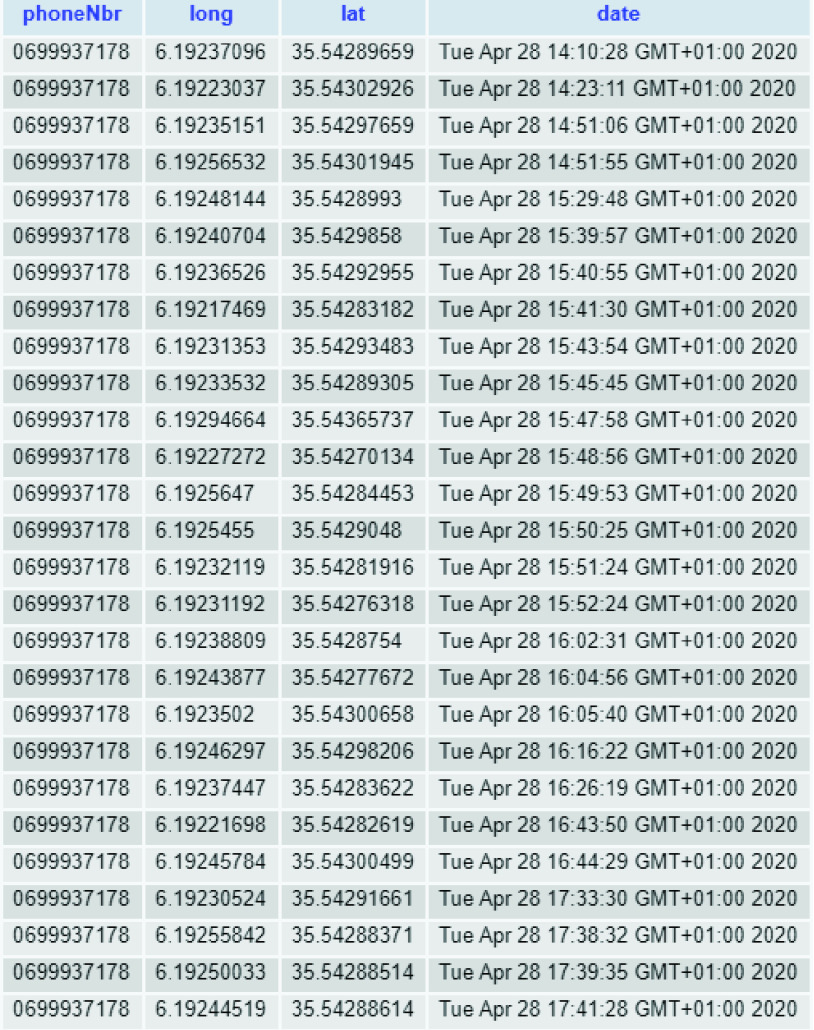
Snippet of the collected trajectories of one user.

The investigation system is continuously fed with both the locations of users and health data (e.g., confirmed patients, closed cases, etc.). Figure 12 provides an example of historical trajectories taken by a single user of the system. As previously stated in the paper, the collected locations of persons are stored as immutable append-only data (since there is no need to change the gathered tracks, no updates are allowed).

Due to the continuous periodic data gathering, an important storing space is needed. However, for demonstration, in this small-scale evaluation version, a single PC was considered to store both health and localization data.

### C. Authorities Interface

Numerous functionalities can be offered to the government, health authorities, and other entities that are responsible for monitoring, evaluating, and controlling the outbreak. The most important functionality is the ability to search for novel suspected cases. To do so, the authorities must first provide the investigation system with the necessary new coronavirus cases, deaths, and recovered patients.

**FIGURE 13.**
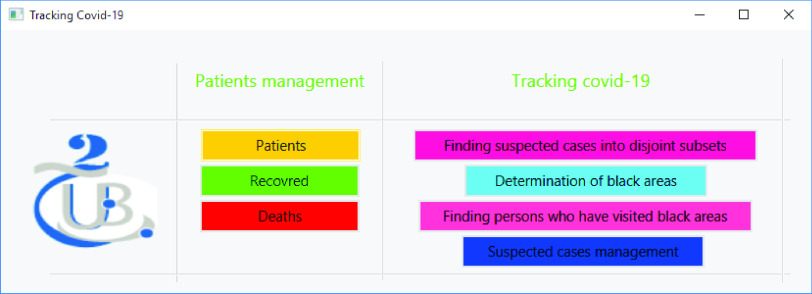
Main application interface for authorities.

Figure 13 shows the main interface of the developed application. We distinguish two categories of activities: (i) on the left side, we find tasks related to patient management, and (ii) on the right side, activities related to virus tracking. Upon discovering/entering newly infected cases, the proposed system can: (1) find all people who had contact with these newly infected patients, (2) determine black areas, (3) find persons who visited black areas, (4) and manage/monitor suspected cases to ensure that they are respecting the imposed quarantine.

**FIGURE 14.**
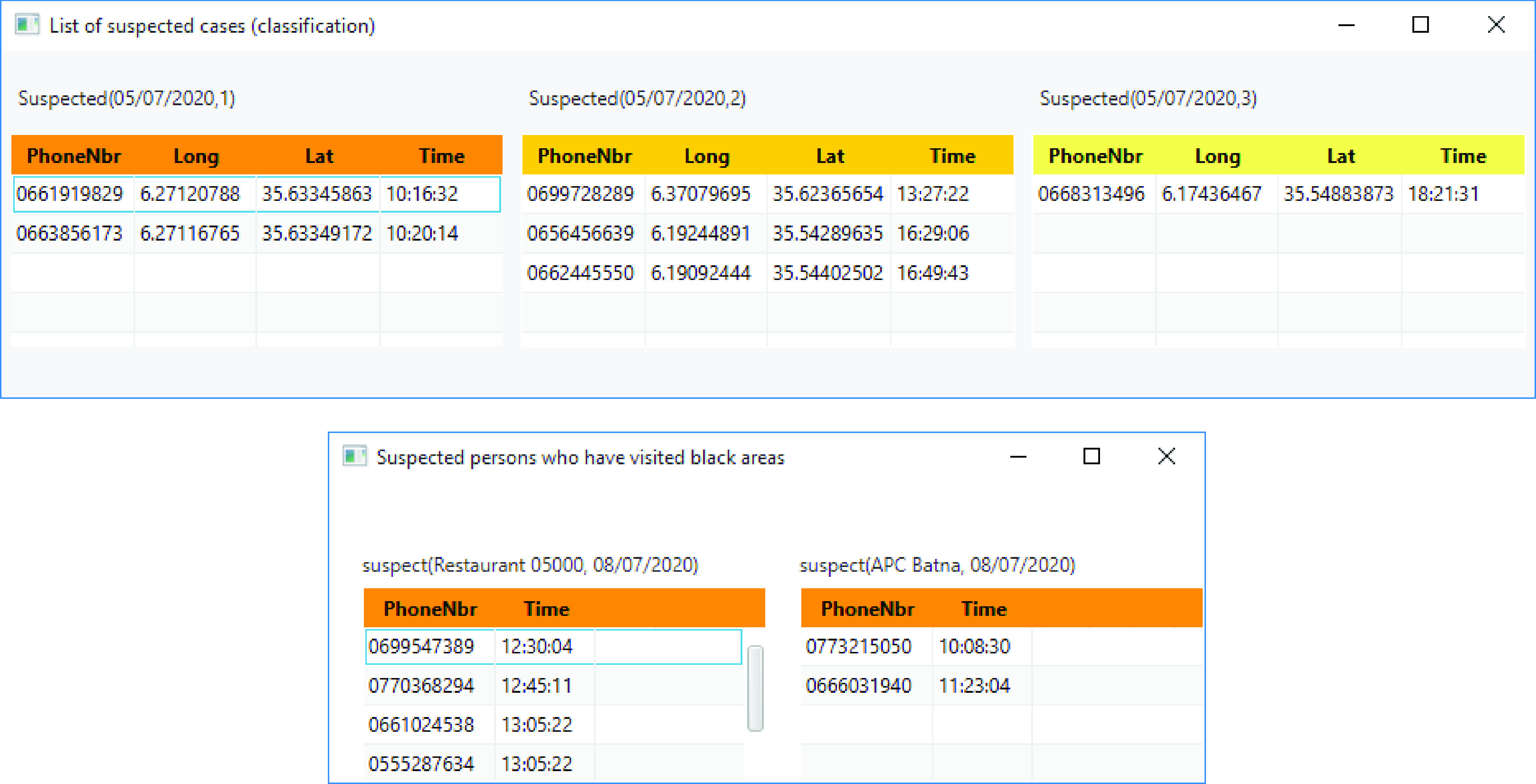
Examples of interfaces for government and health authorities.

As previously explained, the proposed system classifies the newfound suspected people into disjoint subsets (top part of Figure 14). For example, if we assume that the newly discovered patients belong to subset $S_{0}$, then, all persons who had direct contact with them will belong to $S_{1}$. And, all persons who had direct contact with persons from $S_{1}$ will belong to $S_{2}$, and so forth. This way, categories of high and low risk can be easily determined. Once determined, all suspected cases will be (1) stored in the system and (2) informed to immediately start self-isolation at home. The bottom part of Figure 14 shows how the system can identify both black areas and persons who have frequented them.

The investigation system must be designed so that (1) it helps the authorities in their endeavor of fighting coronavirus, and (2) it protects the privacy of users. The latter goal can be achieved through the designed GUI and offered functionalities, which must be defined based on a well-established privacy model.

## IV. On the Mathematical Modeling and Prediction of Infectious Diseases

Mathematical modeling of infectious diseases is a powerful tool that allows analyzing, understanding, and predicting the behavior of pandemics [14]–[16]. For instance, these models can be utilized to help authorities set up the best strategies for successful pandemics control. In the following three subsections, we will briefly show how different mathematical prediction models can benefit from the proposed investigation system. More exactly, how can the two generated lists/sets (SUSPECTED_*t*_ and SUSPECT_*t*_) of (1) suspected/infected persons and (2) foci of infection (black areas) help attain a more accurate realistic estimate of disease spreading rate.

### A. $\theta $-SEIHRD Model

More recently, a new mathematical model, called $\theta $-SEIHRD,^4^ has been specifically proposed for the coronavirus disease [17]. This model assumes that the pandemic spatial distribution inside a territory is omitted. $\theta $-SEIHRD also assumes that people in a territory are characterized to be in one of the following nine compartments: S (Susceptible), E (Exposed), I (Infectious), Iu (Infectious but undetected), HR (Hospitalized or in quarantine at home), HD (Hospitalized that will die), Rd (Recovered after being previously detected as infectious), Ru (Recovered after being previously infectious but undetected) or finally D (Dead by COVID-19). The most important parameters of this model that determine the pandemic spreading are: $\beta ^{(i)}_{E}$, $\beta ^{(i)}_{I}$, $\beta ^{(i)}_{Iu}$, $\beta ^{(i)}_{HR}$, and $\beta ^{(i)}_{HD}$. These parameters represent the disease contact rates ($day^{-1}$) of a person in both the corresponding compartments ($E$, $I$, $Iu$,…) and territory $i$ (without taking into account the control measurements). Indeed, it is very hard to determine these contacts rate without having an idea about who contacted whom. Also, the contacts between persons are not sufficient to estimate the coronavirus contact rates. This disease can be transmitted without direct contact with patients (e.g., touching an object previously used by an infected person). The determination of both sets SUSPECTED_*t*_ and SUSPECT_*t*_ helps to accurately estimate the contacts rate and to determine the set of *Infectious but undetected* (Iu) persons. In addition to this, our proposed investigation system can also help to determine the pandemic spatial distribution inside any monitored territory. As it is well-known, it is very important to understand diseases spreading, determine the safe and risky areas, and take control measurements with insight.^4^$\theta $-SEIHRD: Susceptible Exposed Infectious Hospitalized Recovered Dead. $\theta $ represents the fraction of infected people that has been detected.

### B. SIR Model

SIR^5^ is one of the most studied mathematical models for the spreading of infectious diseases [15], [16], [18]. In this model, the most important parameters that determine the pandemic spreading are $\alpha $, $\gamma $, and $\beta $. Where $\alpha $ is the probability of becoming infected, $\gamma $ is the number of infected people (new infections are the result of contact between infectives and susceptibles), and $\beta $ is the average number of transmissions from an infected person (determined by the chance of contact and the probability of disease transmission). The major difficulty or drawback of this model is estimating these parameters. The answer to this question is our proposed investigation system.^5^SIR: Susceptible Infective Removed model.

### C. SEIR Model

SEIR^6^ is also one of the most important mathematical models for the spreading of infectious diseases [19]. In this model, the main parameter that determines the pandemic spreading is the contact rate $\beta (t)$, which is the average number of susceptibles in a given population contacted per infective per unit time. The main difference between SEIR and SIR lies in the addition of a latency period. More details can be found in [19]–[22]. Endowing the SEIR model with accurate contact information can help it obtain better results about the spreading (prediction) of infectious diseases. As previously highlighted, this is exactly where our proposed solution comes in handy. It can help to understand and estimate important parameters for numerous mathematical models proposed for infectious diseases.^6^SEIR: Susceptible Exposed Infectious Recovered model.

## V. System Advantages and Shortcomings

In this section, we provide the main advantages and disadvantages of the proposed investigation system. First, among the numerous interesting features of this IoT system we mainly cite:

• This system allows tracking the trajectories of infected persons, and this, days before the appearance of their symptoms. The system also allows determining all persons who were in close contact with infected patients. Thus, the former can be immediately confined and the proper measurements can be rapidly taken by health authorities. Since all persons who have met infected patients can be identified earlier (i.e., parents, friends, colleagues, and most importantly, those who cannot be easily identified using conventional techniques), the proposed tracking system allows an early outbreak control.
• The proposed system can be utilized to identify black zones, which can be the main source of virus-spreading. As previously explained, a given location is said to be a source of contamination if numerous infected persons have visited it (the intersection zones of high infections can be identified based on the trajectories of infected persons).
• According to [2], the coronavirus can live for several hours without a host. To remedy this, the proposed system can be configured to find all uninfected persons who have visited locations that were previously visited by other infected persons (and this, hours after the patients have left this location).
• The proposed system can help reduce the economic damages generated by the suspension of all activities. Instead of shutting down all sports, social and economic activities, the authorities can impose confinement on only a few people.
• In addition to coronavirus, the proposed solution can be utilized for any potential outbreak which might be more powerful and have a higher spreading pace.

Despite its advantages, the proposed solution suffers from some issues that must be adequately tackled:

• For instance, if certain persons do not respect the safety instructions (e.g., they did not intentionally or unintentionally save their trajectories when leaving their houses or cars), it will be impossible to determine whether they have met infected persons. The absence of one or several trajectories does not mean the total failure of the proposed investigation system. Indeed, the efficiency of this system is proportional to its users. If $x$% of the population has been involved, then approximately $x$% of the persons who had close contact with patients will be determined.
• The proposed system can be empowered with extra functionalities such as machine/deep learning abilities. Moreover, the system can be utilized to determine whether citizens are respecting social distancing and also to check whether the persons who are suspected to be infected are respecting the imposed confinement.
• Based on the gathered geolocation data, numerous relevant information can be extracted (e.g., the distance between people, large events, movement speed, etc.). In fact, in addition to this, IoT devices allow getting other important information like people’s temperature. One way to achieve this goal is to combine the proposed solution with BANs (Body Area Networks). For instance, using special bracelets that can collect geolocation coordinates along with the temperature of its user. Considering such BAN devices will certainly make the tracking system more efficient (it provides important additional public health information and allows detecting more suspected/infected persons).
• Finally, for a more efficient tracing system, other limitations/issues also still need to be addressed. For example, (1) how can one guarantee the privacy of people, and (2) deal with battery depletion of mobile devices, particularly with the use of GPS.

## VI. Related Work

To limit the coronavirus propagation, numerous countries have released recently-developed applications for COVID-19 tracking. As examples, we mention, China, South Korea, India [23], Russia [24], Italy, Norway, France, Germany [25], Australia [26], and Canada. Indeed, while these countries resorted to the help of Information technologies and applications, others have refrained from using them due to several concerns (e.g., privacy, etc.).

Generally speaking, the released applications are used to ensure three kinds of tasks: (1) provide information/updates about the pandemic, (2) record any close contacts between end-users, and (3) monitor persons who are currently quarantined (to check if they are following the instructions and respecting the imposed self-isolation). The collected information (recorded close contacts,…) is exploited when new persons test positive (anyone who had close contact with a patient will be informed to start self-isolation).

To record close contacts with patients, two techniques have been followed; namely the centralized and decentralized approaches. In the former (which, to the extent of our knowledge, is the most adopted one), geolocalization (GPS) data is saved in a centralized manner, giving thus health authorities a global view of the status quo. In the second approach, the collected data is saved by users’ smartphones. That is, each smartphone detects all other smartphones that are in its proximity. In fact, in this case, since BLE technology^7^ is utilized, no need for (1) geographic localization, or (2) saving data in a centralized fashion. More details about these two points (applications taxonomy, etc.) can be found in [27].^7^BLE: Bluetooth Low Energy.

Even though many countries have launched their proper information systems to trace and slowdown the coronavirus [23]–[26], to the best of our knowledge, no research works have been dedicated to deeply study such systems from the theoretical and technical points of view. Indeed, most of the existing studies deal with contact-tracking systems but only from an abstract, statistical or taxonomical perspective [27]–[31]. With that in mind, this work has been proposed to cover this gap and to provide details about this issue to facilitate future developments of efficient tracking tools.

## VII. Conclusion and Recommendations

In this work, we proposed a system that can quickly determine all persons who are suspected to be infected by the coronavirus. While being specific to this virus, the proposed solution can also be applied to control other pandemics. The objective (as specified in the paper) is not tracking people but tracking extremely dangerous viruses. To ensure a practical and more efficient application of this proposal, the following points must be taken into account:

• *System utilization*: to keep the public health situation under control, it is recommended to launch such an automatic investigation process as early as dangerous rapid infections start taking place. In the case where the outbreak achieves large scales, the operation of detecting, tracking, and surveilling a huge number of infected people might become useless.
• *System efficiency*: for a more efficient system, we recommend exploiting all possible existing resources that can enhance the quality of the collected data (cellphones, security camera footage, credit card records, etc.).
• *Data privacy*: finally, to ensure the privacy and security of the collected data, which is personal and highly sensitive, an authority of trust must manage this whole process.
